# Draft genome sequence of *Priestia flexa* BCA501, a tannin-degrading bacterium isolated from the gut of silver therapon (*Leiopotherapon plumbeus*)

**DOI:** 10.1128/mra.00524-26

**Published:** 2026-06-15

**Authors:** Nico G. Dumandan, Annie Cita T. Kagaoan, Sheryl Lozel B. Arreola, Eldrin D. L. R. Arguelles

**Affiliations:** 1 National Institute of Molecualr Biology and Biotechnology (BIOTECH), University of the Philippines Los Baños, College 700375, Laguna, Philippines; 2 Institute of Chemistry, College of Arts and Sciences, University of the Philippines Los Baños, College 482308, Laguna, Philippines; University of Manitoba, Winnipeg, Canada

**Keywords:** *Priestia flexa*, tannin degradation, gut microbiome, aquafeed bioprocessing

## Abstract

The draft genome sequence of *Priestia flexa* BCA501, isolated from the gut of silver therapon (*Leiopotherapon plumbeus*), is reported. Genome analysis revealed genes associated with tannin degradation, including a putative tannase, indicating its potential application in bioprocessing tannin-rich substrates.

## ANNOUNCEMENT

Tannins are plant polyphenols that reduce aquafeed digestibility by binding proteins and digestive enzymes (1). Because these antinutritional compounds are common in plant-derived feed ingredients, there is increasing interest in isolating tannin-degrading microorganisms for potential application in sustainable aquafeed bioprocessing. Silver therapon (*Leiopotherapon plumbeus*), locally known as ayungin, is a Philippine endemic freshwater fish valued as a food fish (2). Strain BCA501 was isolated from the gut of wild-caught juvenile silver therapon obtained from the Bureau of Fisheries and Aquatic Resources Regional Office No. 5 (Fabrica, Bula, Camarines Sur, Philippines) by enrichment in a defined medium containing tannic acid as the sole carbon source, followed by heat treatment at 65°C for 20 min to select for spore-forming bacteria, and repeated streaking on Luria-Bertani agar until a pure culture was obtained, as previously described (3).

For genomic DNA extraction, BCA501 was cultured in nutrient broth at 37°C for 18 h with shaking at 150 rpm. Cells were harvested by centrifugation, and genomic DNA was extracted using the NucleoSpin Microbial DNA Isolation Kit (Macherey-Nagel, Germany) following the manufacturer’s instructions without modification. The extracted DNA was then submitted to Macrogen Inc. (Seoul, South Korea) for sequencing. A sequencing library was prepared using the TruSeq Nano DNA Library Prep Kit with a 350-bp insert size and sequenced on the Illumina NextSeq X platform to generate 150-bp paired-end reads. A total of 22,383,311 paired-end reads were generated. Raw reads were quality-assessed using FastQC v0.12.1 (4), and no trimming was performed due to the high overall read quality (Q30 ≥90.9%). Taxonomic assignment of strain BCA501 was determined based on whole-genome comparisons and genome annotation analyses, which are established approaches for prokaryotic species delineation and classification (5, 6).

*De novo* genome assembly was carried out using SPAdes v4.2.0 (7), followed by iterative polishing with Pilon v1.24 (8). Assembly quality metrics were evaluated using QUAST v5.3.0 (9). Genome annotation and coding sequence prediction were performed using Prokka v1.14.6 (10), and functional classification of predicted proteins was conducted using eggNOG-mapper v5.0.2 (11) against the EggNOG Database v5. Genome completeness and contamination were assessed using CheckM v1.2.5 (12). BUSCO v5.8.3 analysis (13) using the bacteria_odb10 data set indicated 100.0% completeness (98.4% single-copy and 1.6% duplicated), with no fragmented or missing BUSCOs. Key genome assembly, quality, and annotation features are summarized in Table 1. All bioinformatic analyses were performed using default parameters unless otherwise stated.

**TABLE 1:** Genome assembly, quality, and annotation features of strain BCA501

Genome features	Value
Assembly statistics^*a*^	
Genome size (bp)	4,122,898
Number of contigs	200
N50 contig length (bp)	42,897
L50 contig	27
Largest contig (bp)	248,552
GC content (%)	37.53
Genome quality^*b*^	
Genome completeness	100
Genome contamination	0.06
Annotation features^*c*^	
Total number of genes	4,463
Total number of coding genes	4,263
Total number of CDS	4,388
Total number of RNA genes	75
rRNAs	2, 2, 5 (5S, 16S, 23S)
tRNAs	61
ncRNAs	5

^*a*^ Derived from QUAST v5.3.0.

^*b*^ Estimated using CheckM v1.2.5.

^*c*^ Derived from NCBI Prokaryotic Genome Annotation Pipeline (PGAP) v6.11.

Genome annotation identified a putative tannase (tannin acyl hydrolase) and several tannase-related genes implicated in phenolic compound metabolism, consistent with the previously demonstrated tannin-degrading activity of BCA501 in liquid culture and solid-state fermentation (1, 14).

## Data Availability

The whole-genome shotgun project has been deposited in GenBank under accession number JBXGSY000000000. The BioProject accession number PRJNA1455349 includes the BioSample (SAMN57318911) and SRA (SRR38146715) records associated with this study.
